# Industrial upgrading and new quality productive forces: evidence from Chinese industrial parks

**DOI:** 10.3389/fpubh.2025.1683019

**Published:** 2025-12-03

**Authors:** Chao Li, Meiyi Wang

**Affiliations:** Changchun Sci-Tech University, Changchun, China

**Keywords:** industrial upgrading, new quality productive forces, industrial parks, probability of informed trading, R&D investment

## Abstract

Industrial parks serve as vital platforms for industrial upgrading and the improvement of corporate productivity. This study hence investigates the impact of industrial upgrading driven by industrial park development on new quality productive forces based on a 2012 to 2022 sample concerning industrial parks located across China's prefecture-level cities. The findings reveal that industrial upgrading markedly boosts firm-level new-quality productivity through decreasing the likelihood of informed trading and facilitating corporate R&D investment. Further analysis confirms both the agglomeration function of establishing industrial parks and the heterogeneous magnitudes of industrial upgrading's boosting effect depending on firm ownership, environmental violation incidents, and city characteristics. Regarding practical implications, this study offers guidance for governments to optimize industrial park policies and for enterprises to improve their new quality productive forces.

## 1 Introduction

With the profound transformation of the global economic structure and the rapid advancement of technological progress, industrial upgrading has become a key driver of high-quality economic development. Industrial parks, as essential platforms for industrial clustering and innovation-driven growth, play a vital role in promoting regional economic development, optimizing resource allocation, and enhancing corporate competitiveness. In recent years, China has actively promoted industrial park development to facilitate the transformation and upgrading of traditional industries and the nurturing of emerging industries. New-type productive forces represent a novel form of productive capacity driven by innovation as its core engine, emerging as a critical factor for enterprises to navigate complex market environments and achieve sustainable development. From the perspective of industrial parks, examining the impact mechanisms of industrial upgrading on the development of enterprises' new-type productive forces holds significant theoretical and practical value.

Industrial upgrading represents the core pathway for industries to achieve optimization through technological transformation and structural advancement. This process involves the efficient reallocation of production factors within the sector, significantly enhancing productivity (1). Industrial parks create a favorable development environment for enterprises by providing infrastructure, technical support, and policy incentives. Driven by industrial parks, enterprises achieve industrial upgrading through technological innovation, digital transformation, and green low-carbon development (2), thereby advancing the development of new productive forces. However, during this upgrading process, enterprises also face a series of challenges, such as technological barriers, resource constraints (3), and market uncertainties (4). These challenges not only impact the short-term operational performance of enterprises but may also significantly influence their long-term competitiveness and capacity for sustainable development. Therefore, examining the impact of industrial park-driven industrial upgrading on the development of new types of productive forces in enterprises holds both theoretical and practical importance.

In fact, during industrial upgrading, whether it involves technological innovation or management model transformation, enterprises are closely linked to the development of new productive forces. On the one hand, industrial upgrading lays the groundwork for new productive forces by promoting technological progress and enhancing production efficiency. Industrial upgrading facilitates energy- and environment-oriented technological progress (5), thereby promoting the development of new productive forces within enterprises. The adoption of smart manufacturing, green technology, and digital platforms not only increases enterprise productivity but also fosters new business models and market opportunities (6).

On the other hand, industrial upgrading also requires enterprises to have higher organizational capabilities and resource allocation skills. If businesses fail to adapt to the changes brought by industrial upgrading, they may face risks such as falling production efficiency and diminished market competitiveness (7). Enterprises with policy advantages in resource allocation can swiftly adapt to changes brought by industrial upgrading and are better positioned to pursue green technological innovation (8). Moreover, as society increasingly emphasizes sustainable development and innovation-driven growth, enterprises must achieve green transformation and breakthroughs in innovation through industrial upgrading to boost their competitiveness in the modern economic environment (9).

Industrial parks, as key catalysts for industrial upgrading, offer enterprises technical, financial, and policy support. They also foster cooperation and resource sharing among enterprises through industrial chain synergy and the development of innovation ecosystems. For example, some industrial parks establish technology innovation platforms and incubators to help enterprises overcome technical bottlenecks and accelerate the emergence of new productive forces (10). However, differences in the development levels and support capabilities of industrial parks can lead to varied opportunities and challenges for enterprises during industrial upgrading. For instance, parks with abundant resources and strong policy backing may better facilitate technological breakthroughs and industrial advancement. Conversely, parks with limited resources and insufficient support may constrain enterprise development potential (11). Therefore, studying the impact of industrial park-driven industrial upgrading on the development of enterprises' new-quality productive forces requires a comprehensive analysis of factors such as park characteristics, enterprise attributes, and external environmental conditions.

Given this, this study selected Chinese A-share listed companies from 2012 to 2022 as its research focus to explore how industrial park-driven industrial upgrading influences the development of enterprises' new-quality productive forces. It investigates the mechanisms of their interaction from the dual perspectives of technological innovation capabilities and resource allocation efficiency. The study found that industrial upgrading driven by industrial parks significantly enhances the development of enterprises' new-quality productive capacity, with a more pronounced effect observed in companies with strong technological innovation capabilities and high resource allocation efficiency. Mechanism analysis indicates that industrial upgrading indirectly promotes the formation of new-quality productive capacity by improving enterprises' technological innovation capabilities and optimizing resource allocation efficiency. Further research suggests that high-tech industrial parks and national-level development zones exert a more substantial positive influence on enterprises' new quality productive forces; in the eastern, central, and western regions, the positive impact of industrial upgrading driven by industrial parks on new quality productive forces shows a gradually weakening trend.

This study aims to contribute to the following aspects: (1) although scholars have produced numerous findings on the impact of industrial upgrading on enterprises' new-quality productive forces (12, 13), exploration of its underlying mechanisms remains insufficient. This paper innovatively examines the role of industrial upgrading in fostering new-quality productive forces from an industrial park perspective, thereby complementing the existing literature. (2) By introducing informed transaction probability and R&D investment as mediating variables, it examines their role in the transmission mechanism between industrial upgrading and new-quality productivity, deepening our understanding of how industrial upgrading enhances corporate new-quality productivity. (3) The study reveals regional heterogeneity in the impact of industrial upgrading on new-quality productivity development, providing a basis for governments to formulate differentiated policies.

The remaining content of this paper is organized as follows. Section 2 presents a literature review and research hypotheses. Section 3 outlines the research design, including data sources, variable definitions, and model construction. Section 4 provides empirical results and analysis, such as benchmark regression, endogeneity tests, robustness checks, mechanisms of action, moderation effects, and heterogeneity analysis; and the final section concludes with research findings and implications.

## 2 Literature review and hypothesis

### 2.1 Related research on industrial upgrading

Industrial upgrading, as an important topic in the fields of economics, management, and development studies, has attracted considerable attention in recent years. It generally refers to the process of shifting industries from low-value-added to high-value-added sectors and from labor-intensive to technology-intensive sectors through technological innovation, structural adjustments, and efficiency enhancements (14). This process not only involves technological advancement and efficiency gains within industries but also includes extending industrial chains, restructuring value chains, and optimizing industrial frameworks (15). As globalization and technological revolutions deepen, the concept of industrial upgrading has grown from a sole economic growth goal to a multidimensional pursuit that includes sustainable development, green transformation, and innovation-driven growth (16).

In contemporary industrial upgrading research, technological innovation is seen as the key force driving industrial advancement. It not only includes the research, development, and application of new technologies but also involves technology diffusion, integration, and the significant influence of technology on production methods and business models (17). The broad adoption of digital technologies, artificial intelligence, and green innovations is transforming the production processes and value chain layout of traditional industries (18). Additionally, industrial upgrading highlights the coordinated growth of industrial chains and the synergistic effects within regional economies. Through vertical integration and horizontal expansion of these chains, companies can achieve efficient resource distribution and a comprehensive boost in competitiveness (19, 20).

At the policy level, industrial upgrading is seen as a crucial route to achieving high-quality economic growth. Governments across the globe are encouraging industrial upgrading by developing industrial policies, offering financial support, and enhancing innovation ecosystems (21). Additionally, industrial parks, as key platforms for industrial upgrading, foster a supportive environment for enterprises by providing infrastructure, technical assistance, and policy incentives (22). Nonetheless, the process of industrial upgrading confronts many challenges, such as technological hurdles, resource limitations, and market uncertainties, which can impede the success of industrial upgrading (23).

Although the significance of industrial upgrading for economic growth and enterprise competitiveness is widely acknowledged, research on how industrial upgrading influences the development of new-quality productive forces within enterprises remains relatively limited. As a new form of productive forces driven by innovation, the creation and improvement of new-quality productive forces are closely linked to industrial upgrading (24). Existing research has mainly concentrated on the effects of industrial upgrading on economic growth, employment structures, and regional development, with limited focus on its mechanisms of influence on productivity growth at the micro-enterprise level (25). Therefore, investigating the impact of industrial upgrading on new quality productive forces from an enterprise perspective not only enriches our understanding of the economic effects of industrial upgrading but also offers theoretical foundations and practical guidance for enterprises pursuing sustainable development.

### 2.2 Research on the influencing factors of new quality productivity of enterprises

Identifying factors influencing enterprise new quality productive forces constitutes a trending topic (26), involving multiple aspects such as technological innovation, resource allocation, policy environment, and organizational capabilities. Technological innovation is regarded as the key driving force behind the development of new quality productive forces. Through R&D investment, technology adoption, and independent innovation, enterprises can greatly enhance production efficiency, optimize product structures, and explore new market opportunities (27). However, the success of technological innovation is often affected by a company's R&D capacity, technology conversion efficiency, and external technological conditions. Suppose a company lacks adequate resource support or encounters technological obstacles during the innovation process. In that case, it may lead to low innovation efficiency, thus restricting the improvement of new quality productive forces (28).

Resource allocation efficiency is another important factor affecting a company's new quality productive forces. Efficient resource allocation helps companies optimize the use of production factors, lower production costs, and boost output efficiency (29). For instance, by utilizing digital technologies for intelligent supply chain management, companies can better coordinate internal and external resources and improve operational efficiency. However, gains in resource allocation efficiency are often limited by a company's internal management capabilities and external market conditions. If enterprises face information asymmetry or decision-making errors during resource allocation, this can lead to resource wastage and decreased production efficiency (30).

The policy environment also greatly influences the development of enterprises' new quality productive forces. Governments can foster a favorable development environment for businesses by formulating industrial policies, offering financial support, and enhancing the innovation ecosystem (31). The development of high-tech industrial parks and national-level development zones provides companies with technical, financial, and policy backing, thereby encouraging the growth of new quality productive forces. However, variations in the policy environment may lead to different opportunities and challenges for enterprises across regions. Areas with abundant resources and strong policy support are more likely to facilitate technological breakthroughs and productivity gains. Conversely, regions with limited resources and weaker support may restrict the development potential of enterprises (32).

Additionally, organizational capabilities are also key factors affecting the level of new-type productive forces in enterprises. These capabilities include management, innovation, and adaptability, which determine whether an enterprise can effectively combine internal and external resources and respond to market changes (33). By establishing flat organizational structures and flexible management mechanisms, enterprises can respond more swiftly to market demands and achieve technological breakthroughs. However, improving organizational capabilities often requires long-term development and optimization. If a company does not invest enough in developing organizational capabilities, it may hinder the growth of its new quality productive forces (34).

Although the importance of new quality productive forces for high-quality corporate development has been widely recognized, research on its influencing factors remains relatively limited. Existing studies have primarily focused on single dimensions such as technological innovation and resource allocation. At the same time, few have explored the interactions among various factors and their comprehensive impact on new quality productive forces from a systemic perspective (35). Therefore, conducting research on the influencing factors of corporate new quality productive forces from a multidimensional and multi-level perspective not only deepens our understanding of the formation mechanisms of new quality productive forces but also provides theoretical foundations and practical guidance for enterprises to achieve sustainable development.

### 2.3 Theoretical analysis and research hypotheses

#### 2.3.1 The impact of industrial upgrading on the enterprise's new quality productive forces level

Industrial upgrading, as a key pathway for advancing high-quality economic growth, significantly influences the development and improvement of new quality productive forces levels within enterprises. This form of productivity, driven by innovation, relies on various factors such as technological progress, optimized resource allocation, and strengthened organizational capabilities (36, 37). Industrial upgrading offers vital support for boosting enterprises' new quality productive forces capacity through technological innovation, supply chain restructuring, and efficiency enhancements.

From the perspective of technological innovation, industrial upgrading promotes the development of high-quality productive capacity through technological advancements. Technological innovation serves as the core driver of industrial upgrading and a key component in building high-quality productive capacity (38). During this process, enterprises can significantly improve production efficiency, refine product structures, and explore new market opportunities through R&D investments, technology adoption, and independent innovation. The implementation of intelligent manufacturing, green technologies, and digital platforms not only enhances enterprise productivity but also fosters new business models and market prospects (39).

From a resource optimization perspective, industrial upgrading improves the level of high-quality productive forces via better resource allocation. Effective resource allocation enables enterprises to optimize the combination of production factors, cut production costs, and boost output efficiency. By pursuing vertical integration and horizontal expansion within the industrial chain, enterprises can achieve efficient resource distribution and strengthen overall competitiveness. The utilization of digital technologies to manage intelligent supply chains allows enterprises to coordinate internal and external resources more effectively and improve operational efficiency (40).

From an organizational capability's standpoint, industrial upgrading advances the creation of high-quality productivity by strengthening organizational capabilities. Organizational capabilities are vital skills that enable enterprises to integrate internal and external resources and adapt swiftly to market changes (41). Through establishing flat organizational structures and flexible management systems, enterprises can respond more swiftly to market demands and attain technological breakthroughs. Developing technology innovation platforms and incubators accelerates technology transfer and application, thereby elevating high-quality productive capacity (42).

From a policy environment perspective, industrial upgrading promotes the development of high-quality productive capacity through better policy optimization. Governments can create a favorable environment for businesses by implementing industrial policies, providing financial support, and strengthening the innovation ecosystem (43). The establishment of high-tech industrial parks and national-level development zones offers enterprises technical, financial, and policy assistance, which encourages the growth of high-quality productivity (44, 45). As a result, industrial upgrading provides external safeguards for the development of high-quality productivity in enterprises by improving the policy environment.

Based on the above theoretical analysis, this paper proposes the following research hypothesis:

H1: Industrial upgrading has a significant positive effect on the level of new-type productive forces in enterprises; that is, the greater the degree of industrial upgrading, the higher the level of new-type productive forces in enterprises.

#### 2.3.2 Analysis of the underlying mechanism

As a key means of promoting high-quality development in enterprises, industrial upgrading not only enhances the technical level and production efficiency but also indirectly fosters the formation of new-type productive forces by optimizing the information environment and reducing information asymmetry. New quality productive forces, driven by innovation, represent a new form of productivity whose growth relies on multiple factors such as technological progress, optimized resource allocation, and improved organizational capabilities (46). Industrial upgrading decreases the likelihood of informed traders, thereby creating a fairer and more transparent market environment for the development of new quality productive forces in enterprises.

On the one hand, information asymmetry is a primary cause of the high likelihood of informed traders (47). During industrial upgrading, enterprises can significantly enhance information transparency and disclosure quality through technological innovation and digital transformation. By introducing advanced information management systems and digital platforms, enterprises can monitor and disclose production and operational data in real time, thereby reducing barriers to information access (19, 20). This improved transparency allows market participants to access information more fairly, decreasing the information advantage of informed traders and thus lowering the likelihood of informed trading (48).

On the other hand, industrial upgrading can strengthen a company's market competitiveness and risk-resistance capabilities by optimizing resource allocation and improving production efficiency. In a highly competitive market environment, companies can better cope with market fluctuations and external shocks through technological breakthroughs and efficiency gains achieved via industrial upgrading, thereby reducing reliance on internal information (49). Through vertical integration and horizontal expansion of the industrial chain, enterprises can attain efficient resource allocation and a comprehensive enhancement of competitiveness, reducing dependence on a single market or technology, and thereby decreasing the information advantage held by informed traders. Additionally, industrial upgrading fosters compliant operations and sustainable development, enhancing enterprises' sense of social responsibility and public image (50). As global attention to sustainable development and innovation-driven growth continues to increase, companies must pursue green transformation and innovation breakthroughs through industrial upgrading to enhance their competitiveness within the new economic environment.

The reduction in the likelihood of informed traders can foster a fairer and more transparent market environment, supporting the development of companies' new productive forces. Firstly, enhanced information transparency can lower companies' financing costs, enabling greater financial support for technological innovation and productivity improvement (51). Secondly, efficient resource allocation can boost corporate production efficiency, establishing a foundation for the emergence of high-quality productivity (52). Lastly, compliant operations and sustainable development can strengthen corporate social reputation and market competitiveness, providing long-term protection for the advancement of high-quality productivity.

H2: The greater the degree of industrial upgrading, the lower the probability of informed trading, thereby improving the level of high-quality productivity.

Industrial upgrading is the process by which enterprises shift from low-value-added to high-value-added production through technological innovation, structural adjustments, and efficiency improvements. In this process, R&D investment acts as the core catalyst for technological innovation and significantly influences the enterprise's production efficiency, product competitiveness, and market position. New quality productive forces are an innovative form of productivity whose development relies on various factors such as technological progress, optimized resource allocation, and improved organizational capabilities.

The core of industrial upgrading lies in technological innovation, and R&D investment is a vital safeguard for this process. During industrial upgrading, enterprises can speed up the research and application of new technologies, improve production efficiency, and enhance product competitiveness by increasing R&D investment. By adopting smart manufacturing, green technology, and digital platforms through R&D efforts, enterprises can not only boost efficiency but also develop new business models and market opportunities (53). Such technological innovation provides the foundation for the development of enterprises' new productive capacities. Industrial upgrading optimizes resource allocation, thereby raising the efficiency and output of R&D investment.

Through vertical integration and horizontal expansion of the industrial chain, enterprises can achieve efficient resource allocation and strengthen overall competitiveness. This optimization of resource allocation offers the necessary support for developing new, high-quality, productive capacities. Furthermore, industrial upgrading encourages compliant operations and sustainable development, which in turn enhances the sustainability of R&D investments (54). As global focus on sustainable development and innovation-driven growth increases, enterprises must pursue green transformation and breakthrough innovations through industrial upgrading to bolster their competitiveness in the evolving economic landscape. By engaging in green technology innovation and sustainable development initiatives, enterprises can improve their social responsibility and public profile, attracting long-term investors and securing ongoing financial backing for R&D. Such compliant practices and sustainable growth provide long-term assurance for the advancement of enterprises' new productive forces.

Increased R&D investment can provide technological support and foster innovative momentum for the development of new productive forces in enterprises. First, increased R&D investment can accelerate the research, development, and application of new technologies, thereby improving production efficiency and product competitiveness (55). Second, increased R&D investment can optimize resource allocation, enhance R&D efficiency, and boost output (56). Finally, increased R&D investment can promote corporate compliance and sustainable development, offering continuous financial backing for R&D efforts.

H3: The higher the level of industrial upgrading, the greater the R&D investment, ultimately improving the level of new productive capacity.

## 3 Data, variables, and methodology

### 3.1 Data and samples

This paper uses data from A-share listed companies from 2012 to 2022 as the analysis sample. After excluding financial industry companies and listed companies with missing financial data, as well as listed companies designated as ST or ^*^ST during the sample period, the final sample spans a period of *T* = 11. The number of industrial parks in this study is obtained from the China Development Zone Association's official website. Industrial upgrading data is sourced from the China Urban Statistical Yearbook. Other data is collected from CSMAR and the Wind database.

### 3.2 Variables

#### 3.2.1 Level of new quality productive forces

Technological innovation is vital for productivity development. New quality productive forces not only emphasize quantitative growth but also highlight their importance as a tool for evaluating technological levels and process management efficiency. Building on this idea, this study adopts the research methodology of Yue et al. (57) and the two-factor theory of productivity, considering the role and value of the labor object in the production process. The entropy method is utilized to determine the weights of each influencing factor, which helps construct an evaluation index system for enterprise new-type productivity. Specifically, the weights are first calculated using the entropy value method, then multiplied by the standardized values of positive indicators and summed. The total is finally multiplied by 1,000 to produce the comprehensive score reflecting the enterprise's level of new-type productivity. Table 1 summarizes the components that constitute the index used to measure new quality productive forces.

**Table 1:** New productivity indicators for enterprises.

**Factor**	**Sub-factor**	**Indicator**	**Indicator value explanation**	**Weight**
Labor	Active labor	Proportion of R&D personnel salaries	R&D expenses – Salaries and wages/operating income	0.290
		Proportion of R&D personnel	Number of R&D personnel/number of employees	0.024
		Proportion of highly educated personnel	Number of employees with a bachelor's degree or higher/Total number of employees	0.040
	Materialized labor (labor objects)	Proportion of fixed assets	Fixed assets/Total assets	0.012
		Manufacturing expense ratio	(Operating activities cash outflow subtotal + Fixed asset depreciation + Intangible asset amortization + Impairment provisions – Cash paid for purchases of goods and services – Wages paid to employees and for employees)/(Operating activities cash outflow subtotal + Fixed asset depreciation + Intangible asset amortization + Impairment provisions)	0.00002
Production tools	Hard technology	R&D expenses – depreciation and amortization ratio	R&D expenses – Depreciation and amortization/revenue	0.345
		R&D expenses – Lease expenses ratio	R&D expenses – Lease expenses/Revenue	0.005
		R&D expenses direct investment ratio	R&D expenses – Direct investment/Revenue	0.256
		Intangible assets ratio	Intangible Assets/Total Assets	0.020
	Soft technology	Total asset turnover ratio	Revenue/Average Total Assets	0.008
		Equity multiplier inverse	Equity/Total assets	0.000001
New quality productive forces				100

#### 3.2.2 Industrial upgrading

Industrial Upgrading Index (*IUI*_*i, t*_): B: based on the research of Wang et al. (58), this paper adopts the weighted output value ratio method to construct the industrial upgrading index, which measures the structural optimization and upgrading level of enterprises across different industrial tiers. Specifically, the industrial upgrading index is calculated using the following formula:


$$\begin{array}{c} IUI_{i,t}=1\times \;primary\;industry\;output\;share_{i,t}+2\\ \times \;secondary\;industry\;output\;share_{i,t}\\ +3\times \;tertiary\;industry\;output\;share_{i,t} \end{array}$$ (1)

The output shares of the primary, secondary, and tertiary industries reflect the distribution of economic activities in traditional agriculture, industry, and services. The design of the weighting coefficients (1, 2, 3) is based on the theoretical framework of industrial upgrading, which suggests that the transition from low-value-added primary industries to high-value-added secondary and tertiary industries is the core feature of industrial upgrading. When an enterprise has a high industrial upgrading index, it indicates that its economic activities in the secondary and tertiary industries constitute a larger proportion, and its level of industrial upgrading is high; conversely, it shows that the enterprise still mainly engages in traditional primary industry activities, and its level of industrial upgrading is low.

#### 3.2.3 Control variables

The following variables were controlled in this study: book-to-market ratio (*BM*_*i, t*_), the ratio of total assets to total market value; return on assets (*ROA*_*i, t*_), the ratio of net profit to total assets; financial leverage ratio (*LEV*_*i, t*_), the ratio of liabilities to total assets; firm age (*AGE*_*i, t*_), the difference between the observation year and the year of firm establishment; firm size (*SIZE*_*i, t*_), the natural logarithm of total assets at the end of the period; Sales growth rate (*ORG*_*i, t*_), the ratio of the difference between current-year revenue and the previous year's revenue to the previous year's revenue; Institutional investor ownership ratio (*INI*_*i, t*_), the proportion of shares held by institutional investors relative to the total shares of the listed company; major shareholder holding ratio (*FIR*_*i, t*_): the ratio of the total number of shares held by the top ten shareholders to the total number of shares. Descriptive statistics for the main variables are shown in Table 2.

**Table 2:** Description of the main variables.

**Variables**	**#Obs**.	**Mean**	**S.D**.	**Min**	**Mdn**	**Max**
*NPRO*	35,404	6.874	3.498	0.287	6.726	19.674
*IUI*	29,531	2.533	0.164	1.900	2.535	2.836
*AGE*	28,653	11.418	7.312	2.000	10.000	28.000
*SIZE*	28,384	22.312	1.220	19.240	22.160	26.130
*BM*	33,520	0.622	0.238	0.115	0.623	1.169
*LEV*	34,979	0.416	0.204	0.053	0.404	1.112
*ROA*	34,840	0.042	0.063	−0.315	0.041	0.234
*ORG*	34,906	0.146	0.345	−0.677	0.099	3.114
*INI*	34,810	42.996	24.613	0.117	44.087	93.141
*FIR*	34,763	59.412	15.099	22.340	60.320	95.290
*VPIN*	34,557	0.178	0.022	0.000	0.177	0.277
*RDSS*	30,984	17.913	1.551	7.720	17.891	25.025
*NIP*	31,959	1,379.244	1,240.193	1.000	896.000	3,788.000

#### 3.2.4 Mediating variables

We measure the probability of insider trading (*VPIN*_*i, t*_) with reference to the research by Abad and Yagüe (59). The following *VPIN*_*i, t*_ measurement is used:


$$\begin{array}{l} VPIN_{i,t}=\frac{\left[\left|V_{i}^{S}-V_{i}^{B}\right|\right]}{V} \end{array}$$ (2)

Here, $V_{i}^{S}$ and $V_{i}^{B}$ respectively represent the selling volume and buying volume of the *i*-th trading volume bucket, and *V* is the transaction volume of each volume bucket.

Which more effectively measures order flow toxicity in quote-driven markets, i.e., the adverse selection risk faced by non-insider traders when providing buy and sell quotes. Specifically, when the *VPIN*_*i, t*_ value for a given trading day is high, it indicates a higher probability of informed trading in the market, which in turn increases order flow toxicity and may lead to a significant increase in market volatility on subsequent trading days.

Total R&D Investment (*RDSS*_*i, t*_): This metric measures the scale of a company's resource commitment to technological innovation by considering the total funds invested in R&D activities over a defined period, usually one year. Total R&D investment thoroughly reflects the level of financial backing for R&D efforts and serves as a vital indicator for evaluating a company's technological innovation capacity and future growth potential. Specifically, when a company's total R&D investment is high, it signifies that the company has allocated substantial funds to R&D activities and may be more inclined to improve production efficiency, optimize product structures, and explore new market opportunities through large-scale technological innovation.

#### 3.2.5 Moderating variables

Industrial Park Location Data (*NIP*_*i, t*_): This study utilizes spatial distribution data of industrial parks nationwide, combined with their economic attribute indicators, to construct an industrial park location dataset, aiming to comprehensively analyze the spatial layout characteristics of industrial parks and their impact on regional economic development.

### 3.3 Methodology

Based on the assumptions outlined above, the following baseline regression model is constructed:


$$\begin{array}{l} Y_{i,t}\;=\;\beta_{0}\;+\;\beta_{1\;}IUI_{i,t\;}+{\;\beta }_{k}Control_{i,t}\;+\;\gamma_{i}+\;\mu_{i}\;+\;\varepsilon_{i,t} \end{array}$$ (3)

Where *Y*_*i, t*_ is the new quality productivity level of the listed company *i* in year *t*, i.e., new quality productivity forces (*NPRO*_*i, t*_); *IUI*_*i, t*_ is the industrial upgrading performance of the listed company *i* in year *t*; *Control*_*i, t*_ is a set of control variables; the model also controls for provincial fixed effects (γ_*i*_) and industry fixed effects (μ_*i*_); ε_*i, t*_ represents the random disturbance term; the subscripts *i* and *t* represent firms and time, respectively, and all regression coefficients' standard errors are clustered at the firm level.

From the perspectives of insider trading probability and R&D investment intensity, this study analyzes the functional channels through which industrial upgrading could affect the level of new quality productive forces in a firm. Following the extant literature, this paper establishes the following mediating effect model (4):


$$\begin{array}{l} MV_{i,t}\;=\;\beta_{0}\;+\;\beta_{1}IUI_{i,t}\;+\;\beta_{k}Control_{i,t}\;+\;\gamma_{i}\;+\;\mu_{i}\;+\;\varepsilon_{i,t} \end{array}$$ (4)

In Model (4), *MV*_*i, t*_ is the mediating variable. The mediating variables used in this study are *VPIN*_*i, t*_ and *RDSS*_*i, t*_, which respectively represent the probability of insider transactions and R&D investment.

From the perspective of the number of industrial parks in different prefecture-level cities as a moderating variable, this study analyzes the moderating effects of industrial upgrading on the level of new quality productive forces in enterprises. The following moderating effect model (5) is established:


$$\begin{array}{c} NIP_{i,t}=\beta_{0}+\beta_{1}IUI_{i,t}+\beta_{2}NIP_{i,t}{+\beta }_{3}{IUI_{i,t}\times \;NIP_{i,t}+Control}_{i,t}\\ +\gamma_{i}+\mu_{i}+\varepsilon_{i,t} \end{array}$$ (5)

## 4 Empirical results

### 4.1 Baseline model regression results

Table 3 presents the baseline test results on the impact of industrial upgrading on the level of new-quality productive capacity. Columns (1) to (4) present the results of the impact of industrial upgrading on the level of new-type productive capacity. Column (1) does not include province and industry fixed effects, Column (2) includes province fixed effects but not industry fixed effects, Column (3) includes industry fixed effects but not province fixed effects, and Column (4) includes both year and province fixed effects. The results show that the estimated coefficients for industrial upgrading as measured by *IUI*_*i, t*_ in columns (1) to (4) are 5.420, 8.458, 5.000, and 7.909, respectively, all of which are significantly positive at the 1% level. This indicates that strong industrial upgrading performance contributes to enhancing a firm's level of new quality productivity. This finding is consistent with the research of Liang et al. (60), supporting Hypothesis 1 of this paper.

**Table 3:** Results of the baseline regression model.

**Variables**	**(1)**	**(2)**	**(3)**	**(4)**
	*NPRO*_*i, t*_			
*IUI*_*i, t*_	5.420^***^	8.458^***^	5.000^***^	7.910^***^
	(16.31)	(17.79)	(17.05)	(18.61)
*AGE*_*i, t*_	0.060^***^	0.035^***^	0.066^***^	0.045^***^
	(7.06)	(4.02)	(8.70)	(5.74)
*SIZE*_*i, t*_	0.761^***^	0.729^***^	0.703^***^	0.664^***^
	(11.53)	(11.07)	(12.26)	(11.59)
*BM*_*i, t*_	−1.941^***^	−1.953^***^	−1.812^***^	−1.821^***^
	(−15.64)	(−15.67)	(−15.19)	(−15.24)
*LEV*_*i, t*_	−1.623^***^	−1.527^***^	−1.038^***^	−0.957^***^
	(−5.99)	(−5.64)	(−4.07)	(−3.77)
*ROA*_*i, t*_	−5.338^***^	−5.335^***^	−4.914^***^	−4.900^***^
	(−12.38)	(−12.33)	(−11.68)	(−11.64)
*ORG*_*i, t*_	0.109^**^	0.109^**^	0.083	0.083
	(2.09)	(2.08)	(1.60)	(1.60)
*INI*_*i, t*_	−0.022^***^	−0.020^***^	−0.016^***^	−0.015^***^
	(−8.45)	(−7.56)	(−6.87)	(−6.07)
*FIR*_*i, t*_	−0.013^***^	−0.015^***^	−0.010^***^	−0.012^***^
	(−3.21)	(−3.74)	(−2.67)	(−3.26)
*Controls*	Yes	Yes	Yes	Yes
Constant	−20.294^***^	−28.760^***^	−19.334^***^	−27.000^***^
	(−13.35)	(−16.08)	(−14.00)	(−16.32)
*Province FE*	No	Yes	No	Yes
*Industry FE*	No	No	Yes	Yes
*#Obs*.	21,786	21,786	21,786	21,786
*R^2^*	0.192	0.192	0.205	0.207

This table reports the results of the impact of industrial upgrading performance on the new quality productivity level, the baseline regression is:*Y*_*i, t*_ = β_0_+β_1_*IUI*_*i, t*_+β_*k*_*Control*_*i, t*_+γ_*i*_+μ_*i*_+ε_*i, t*_, where *Y*_*i, t*_ is the new quality productivity level of listed company i in year t, i.e., *NPRO*_*i, t*_; *IUI*_*i, t*_ is the industrial upgrading performance of listed company i in year t; *Controls*_*i, t*_ indicates control variables in year t, including book-to-market ratio (*BM*_*i, t*_), return on assets (*ROA*_*i, t*_), financial leverage ratio (*LEV*_*i, t*_), firm age (*AGE*_*i, t*_), firm size (*SIZE*_*i, t*_), sales growth rate (*ORG*_*i, t*_), institutional investor ownership ratio (*INI*_*i, t*_), major shareholder holding ratio (*FIR*_*i, t*_). γ_*t*_ and μ_*i*_ denote the province and industry fixed effects, respectively. ε_*t*_ denotes the residual. The t-values of coefficients are given in parentheses.

^**^ and ^***^ represent significance levels of 0.05 and 0.01, respectively.

The primary reason is that industrial upgrading drives productivity progress through technological innovation, introducing advanced technologies, equipment, and processes to enhance production efficiency and product quality, reduce resource consumption, and promote the transformation of production methods toward intelligent and green practices. Industrial upgrading also optimizes resource allocation by phasing out outdated capacity and developing high-value-added industries, enabling enterprises to allocate human, material, and financial resources more reasonably and improve resource utilization efficiency. Additionally, industrial upgrading promotes the optimization of talent structures, drives employee skill enhancement and knowledge updates, and fosters more innovative teams. Finally, industrial upgrading helps expand new markets, develop new products and services, meet market demands, increase market share, and expand revenue sources, significantly enhancing the level of new quality productive forces in enterprises.

### 4.2 Endogeneity test

There may be a complex bidirectional causal relationship between changes in the degree of industrial upgrading of enterprises and their new productive capacity levels. This endogeneity issue may interfere with the research results. To effectively alleviate this issue, this paper adopts an innovative strategy, namely, replacing the original variables with lagged (*L*.*IUI*_*i, t*_) and leading (*F*.*IUI*_*i, t*_) variables of the explanatory variables and re-conducting regression analysis. The test results are shown in Table 3, columns (1) and (2). The regression coefficient of *L*.*IUI*_*i, t*_ on *NPRO*_*i, t*_ is 6.877, and the regression coefficient of *F*.*IUI*_*i, t*_ on *NPRO*_*i, t*_ is 7.831, both of which exhibit a positive relationship at the 1% significance level. This result not only further validates the robustness of the previous conclusions but also provides new perspectives and evidence for a deeper understanding of the complex relationship between corporate industrial upgrading and the level of new productive capacity.

Due to differences in industry attributes, resource endowments, and development strategies among enterprises, not all enterprises implement significant industrial upgrading measures every year of the sample period, which may lead to endogeneity issues caused by sample selection bias. To mitigate this potential issue, this study employs the Heckman two-step method for testing. Following the research approach outlined in relevant studies, this study conducts the following two-step regression: First, a Probit model is used to estimate the selection equation, with the dependent variable being a dummy variable (*dum*_*IUI*_*i, t*_) reflecting the level of *IUI*_*i, t*_ scores. The mean of *IUI*_*i, t*_ is 2.533, so the threshold for *dum*_*IUI*_*i, t*_ is set such that values above the mean are assigned a value of 1, and values below the mean are assigned a value of 0. The explanatory variables include the book-to-market ratio (*BM*_*i, t*_), return on assets (*ROA*_*i, t*_), financial leverage ratio (*LEV*_*i, t*_), firm age (*AGE*_*i, t*_), firm size (*SIZE*_*i, t*_), sales growth rate (*ORG*_*i, t*_), institutional investor ownership ratio (*INI*_*i, t*_), and major shareholder ownership ratio (*FIR*_*i, t*_). Second, the inverse Mills ratio (IMR) estimated from the Probit model is added to the benchmark regression model for re-estimation. Columns (3) and (4) in Table 4 present the results of the two-stage regression. The results show that the regression coefficient in the first stage is 1.013 and is positively significant at the 1% level. After adding the inverse Mills ratio as a control variable, the regression coefficient for industrial upgrading on corporate new quality productive forces is 7.918, and it is positively significant at the 1% level, consistent with the previous conclusions. This indicates that sample selection bias did not significantly affect the research results, further validating the robustness of the research conclusions.

**Table 4:** Endogeneity test results.

**Variables**	**(1)**	**(2)**	**(3)**	**(4)**
	**Granger causality**		**Heckman**	
	*NPRO*_*i, t*_	*NPRO*_*i, t*_	*IUI*_*DUM*_*i, t*_	*NPRO*_*i, t*_
*L*.*IUI*_*i, t*_	6.877^***^		1.013^***^	
	(18.08)		(18.67)	
*F*.*IUI*_*i, t*_		7.831^***^		
		(17.59)		
*IUI*_*i, t*_				7.918^***^
				(18.57)
IMR				−1.7616^*^
				(−1.77)
*Controls*	Yes	Yes	Yes	Yes
Constant				
	/	/	/	/
*Province FE*	Yes	Yes	Yes	Yes
*Industry FE*	Yes	Yes	Yes	Yes
*#Obs*.	19,926	18,675	21,786	21,683
*R* ^2^	0.159	0.228	0.170	0.208

This table presents the results of a series of endogeneity tests. The first stage regression model of Heckman is: *IUI*_*DUM*_*i, t*_ **=** α**+**β_1_*IV*_*i, t*_**+**β_*k*_*Control*_*i, t*_+γ_*i*_+μ_*i*_+ε_*i, t*_, the second stage regression model of Heckman is: *NPRO*_*i, t*_ **=** α**+**β_1_*IUI*_*i, t*_**+**β_*k*_*Control*_*i, t*_+γ_*i*_+μ_*i*_+ε_*i, t*_, where *IV*_*i, t*_ is the instrumental variable, *IUI*_*DUM*_*i, t*_ being a dummy variable reflecting the level of *IUI*_*i, t*_ scores, *NPRO*_*i, t*_ is the new quality productivity level of company i in year t, *IUI*_*i, t*_ is the industrial upgrading performance of listed company i in year t; *Controls*_*i, t*_ indicates control variables in year t, including book-to-market ratio (*BM*_*i, t*_), return on assets (*ROA*_*i, t*_), financial leverage ratio (*LEV*_*i, t*_), firm age (*AGE*_*i, t*_), firm size (*SIZE*_*i, t*_), sales growth rate (*ORG*_*i, t*_), institutional investor ownership ratio (*INI*_*i, t*_), major shareholder holding ratio (*FIR*_*i, t*_). γ_*t*_ and μ_*i*_ denote the province and industry fixed effects, respectively. ε_*t*_ denotes the residual. The t-values of coefficients are given in parentheses.

^*^ and ^***^ represent significance levels of 0.10 and 0.01, respectively.

### 4.3 Robustness test

#### 4.3.1 Replacing the dependent variable

Total Factor Productivity (*TFP*_*OPacf*_*i, t*_) is an important indicator for measuring corporate production efficiency and technological progress, providing a more accurate reflection of the comprehensive utilization efficiency of factors such as technology, capital, and labor in the production process. Conducting robustness testing using *TFP*_*OPacf*_*i, t*_ allows for the evaluation of the model's performance under different production efficiency conditions, thereby enhancing the generalizability and applicability of research findings. Additionally, specific industries or regions may have unique efficiency-influencing factors that could interfere with accurate assessments of the relationship between industrial upgrading and enterprise new quality productive forces. By using *TFP*_*OPacf*_*i, t*_ values, these specific efficiency factors can be excluded, enabling a more accurate assessment of the impact of industrial upgrading driven by industrial parks on enterprise new quality productive forces. Conducting robustness tests using *TFP*_*OPacf*_*i, t*_ can assess whether the model performs consistently under different variables. If the results remain consistent, it indicates that the model has strong robustness, meaning it can provide stable and reliable results for different *TFP*_*OPacf*_*i, t*_ values, thereby enhancing the persuasiveness and credibility of the paper.

As shown in Table 5, Column (1), after regression using *TFP*_*OPacf*_*i, t*_ values, industrial upgrading still has a significant positive impact of 1% on enterprises' new quality productive forces, indicating that industrial upgrading can still effectively enhance enterprises' new quality productive forces, further validating hypothesis H1.

**Table 5:** Robust test.

**Variables**	**(1)**	**(2)**	**(3)**	**(4)**
	**Replace Dep. Var**.	**Replace Indep. Var**.	**Exclude abnormal years**	**Exclude abnormal cities**
	*TFP*_*OPacf*_*i, t*_	*Liquidity*_*i, t*_	*Liquidity*_*i, t*_	*Liquidity*_*i, t*_
*IUI*_*i, t*_	0.445^***^		8.009^***^	6.142^***^
	(5.05)		(19.24)	(11.26)
*AIS*_*i, t*_		1.477^***^		
		(19.18)		
*Controls*	Yes	Yes	Yes	Yes
Constant	−1.823^***^	−11.573^***^	−28.049^***^	−17.392^***^
	(−4.53)	(−9.12)	(−17.12)	(−8.83)
*Province FE*	Yes	Yes	Yes	Yes
*Industry FE*	Yes	Yes	Yes	Yes
*#Obs*.	20,885	21,786	19,237	10,628
*R* ^2^	0.367	0.208	0.206	0.227

This table reports the results of robustness checks, the regression model is: *Y*_*i, t*_ = β_0_+β_1_*X*_*i, t*_+β_*k*_*Control*_*i, t*_+γ_*i*_+μ_*i*_+ε_*i, t*_, where in the Column (1), *Y*_*i, t*_ denotes the total factor productivity, *TFP*_*OPacf*_*i, t*_; in columns (2), *X*_*i, t*_ denotes the indicators of industrial structure upgrading index, *AIS*_*i, t*_; *Controls*_*i, t*_ indicates control variables in year t, including book-to-market ratio (*BM*_*i, t*_), return on assets (*ROA*_*i, t*_), financial leverage ratio (*LEV*_*i, t*_), firm age (*AGE*_*i, t*_), firm size (*SIZE*_*i, t*_), sales growth rate (*ORG*_*i, t*_), institutional investor ownership ratio (*INI*_*i, t*_), major shareholder holding ratio (*FIR*_*i, t*_). γ_*t*_ and μ_*i*_ denote the province and industry fixed effects, respectively. ε_*t*_ denotes the residual. The t-values of coefficients are given in parentheses.

^***^ represent significance levels of 0.01, respectively.

#### 4.3.2 Replacing explanatory variables

To further explore the impact of industrial upgrading driven by industrial parks on enterprises' new quality productive forces, this paper creatively introduces the industrial structure upgrading index. This paper constructs a comprehensive dataset that includes indicators closely related to industrial upgrading, carefully extracted from extensively collected industrial park economic data. Based on this, we further developed an industrial park economic dataset, incorporating the industrial structure upgrading index, which is defined by calculating the ratio of tertiary industry value added to secondary industry value added i.e., Additionally, we established an industrial park technological innovation indicator, measured by the ratio of R&D investment to total industrial park output value.

Column (2) in Table 5 presents the test results after replacing the overall industrial structure upgrade with the industrial structure upgrading index. The regression coefficients for enterprise new quality productive forces are 1.477, and they are significant at least at the 1% level, thereby validating hypothesis H1 once again. This finding not only reinforces previous research conclusions but also provides new evidence from another dimension of industrial structure regarding the association between industrial upgrading and enterprise new quality productive forces, thereby once again validating the correctness of hypothesis H1, which states that the industrial structure upgrading index has a significant impact on enterprise new quality productive forces.

#### 4.3.3 Excluding abnormal years

From an economic perspective, the COVID-19 pandemic that erupted in 2020 undoubtedly caused unprecedented shocks to the global economic system, with its far-reaching impacts and complex economic consequences affecting almost all industries and enterprises. This global public health crisis not only led to a large-scale stagnation of economic activity but also triggered a series of chain reactions, including supply chain disruptions and a sharp decline in market demand, leaving many enterprises facing severe operational pressures and challenges (61). In this context, economic data exhibited high volatility and abnormality, particularly industrial park-related data, which was significantly disrupted, adding considerable complexity to research efforts. Given the high uncertainty that the exceptional year of 2020 has introduced into economic data analysis, this paper excludes the relevant data from 2020 to ensure the accuracy and reliability of the research results. This approach aims to minimize the interference of data from the special period on the overall analysis results, thereby ensuring that the research conclusions more accurately reflect the actual situation and trends under normal economic conditions.

From the regression analysis results in Table 5 (3), we can see that after excluding the data from the exceptional year of 2020, the impact of industrial upgrading driven by industrial parks on enterprises' new productive forces still shows a significant positive relationship of 1%. This result not only validates our hypothesis H1, that industrial upgrading driven by industrial parks can significantly enhance enterprises' new productive forces, but also further reinforces the applicability and stability of this conclusion under normal economic conditions.

#### 4.3.4 Excluding abnormal cities

Among the selected samples, enterprises located in municipalities and provincial capitals may influence the research findings due to the special status and policy advantages of these cities in economic development. These cities typically have more abundant resources, more complete infrastructure, and more intensive policy support, which may lead to significant differences in the impact of industrial park-driven industrial upgrading on enterprises' new quality productive forces compared to other regions. To reduce potential biases caused by these cities, this study excluded sample enterprises from the four municipalities (Beijing, Shanghai, Tianjin, and Chongqing) and 27 provincial capital cities before conducting the regression analysis.

Table 5, Column (4), reports the regression results. The coefficient indicating the impact of overall industrial structure upgrading on enterprises' new quality productive forces is 6.142, and it is significantly positive at the 1% level. This indicates that in other regions of China, industrial park-driven industrial upgrading can also significantly enhance enterprise new quality productive forces. This result not only validates our hypothesis H1, which states that industrial park-driven industrial upgrading has a promotional effect on enterprise new quality productive forces, but also further demonstrates the robustness and universality of this conclusion across different regional contexts.

### 4.4 Mechanism test

In this section, we delve into the intrinsic mechanism through which industrial park-driven industrial upgrading impacts enterprises' new-quality productive capacity. Based on prior theoretical analysis and existing research findings, we find that industrial park-driven industrial upgrading can enhance enterprises' new-quality productive capacity through multiple channels, including reducing the probability of informed transactions and increasing R&D investment. These findings provide important theoretical foundations and practical guidance for industrial parks to drive industrial upgrading and promote high-quality enterprise development.

Table 6, columns (1) and (2), presents the mechanism testing results for the probability of informed transactions (*VPIN*_*i, t*_). In Column (1), the regression coefficient of industrial upgrading (*IUI*_*i, t*_) on the probability of informed transactions (*VPIN*_*i, t*_) is −0.010, and it is significant at the 1% level. In Column (2), the regression coefficient of the probability of informed transactions (*VPIN*_*i, t*_) on the enterprise's new quality productive forces (*NPRO*_*i, t*_) is −19.976, and it is significant at the 1% level. This result indicates that industrial upgrading driven by industrial parks can significantly reduce the probability of informed transactions for enterprises, and the reduced probability of informed transactions plays a significant role in enhancing the enterprise's new quality productive forces. Unlike most previous studies (6), this finding reveals the mediating role of the information environment in the process of industrial upgrading promoting the development of new quality productive forces. This can be explained as follows: industrial upgrading optimizes resource allocation, enhances information transparency and market efficiency, reduces information asymmetry and speculative trading behavior, thereby lowering the probability of insider trading. This improvement in the market environment facilitates enterprises in more efficiently obtaining resources and technology, thereby enhancing new quality productive forces.

**Table 6:** Mechanism analysis results.

**Variables**	**(1)**	**(2)**	**(3)**	**(4)**
	*VPIN*_*i, t*_	*NPRO*_*i, t*_	*RDSS*_*i, t*_	*NPRO*_*i, t*_
*IUI*_*i, t*_	−0.010^***^	7.456^***^	1.074^***^	8.309^***^
	(−7.12)	(17.62)	(7.21)	(19.22)
*VPIN*_*i, t*_		−19.976^***^		
		(−14.92)		
*RDSS*_*i, t*_				0.570^***^
				(15.43)
*Controls*	Yes	Yes	Yes	Yes
Constant	0.458^***^	−17.230^***^	−5.953^***^	−26.307^***^
	(75.63)	(−9.97)	(−9.93)	(−14.85)
*Province FE*	Yes	Yes	Yes	Yes
*Industry FE*	Yes	Yes	Yes	Yes
*#Obs*.	21,752	21,752	18,836	18,836
*R* ^2^	0.271	0.223	0.494	0.251

This table reports the results of the analysis of potential mechanisms by which the industrial upgrading performance affects the new quality productivity level. The first stage of the regression model is: *M*_*i, t*_ **=** α**+**β_1_*IUI*_*i, t*_**+**β_*k*_*Control*_*i, t*_+γ_*i*_+μ_*i*_+ε_*i, t*_, and the second stage of the model is: *NPRO*_*i, t*_ **=** α**+**β_1_*IUI*_*i, t*_**+**_β_2_*Mi, t*_**+**β_*k*_*Control*_*i, t*_+γ_*i*_+μ_*i*_+ε_*i, t*_, where *M*_*i, t*_ is the mediating variable, including the probability of insider trading (*VPIN*_*i, t*_) and total R&D Investment (*RDSS*_*i, t*_), *NPRO*_*i, t*_ is the new quality productivity level of company i in year t; *Controls*_*i, t*_ indicates control variables in year t, including book-to-market ratio (*BM*_*i, t*_), return on assets (*ROA*_*i, t*_), financial leverage ratio (*LEV*_*i, t*_), firm age (*AGE*_*i, t*_), firm size (*SIZE*_*i, t*_), sales growth rate (*ORG*_*i, t*_), institutional investor ownership ratio (*INI*_*i, t*_), major shareholder holding ratio (*FIR*_*i, t*_). γ_*t*_ and μ_*i*_ denote the province and industry fixed effects, respectively. ε_*t*_ denotes the residual. The t-values of coefficients are given in parentheses.

^***^ represent significance levels of 0.01, respectively.

Table 6, columns (3) and (4), presents the results of the mechanism test for R&D investment. In Column (3), the regression coefficient of industrial upgrading (*IUI*_*i, t*_) on R&D investment (*RDSS*_*i, t*_) is 1.074, which is significant at the 1% level. In Column (4), the regression coefficient of R&D investment (*RDSS*_*i, t*_) on firms' new quality productive forces (*NPRO*_*i, t*_) is 0.570, which is significant at the 1% level. This result indicates that industrial park-driven industrial upgrading can significantly increase corporate R&D investment, and that increased R&D investment plays a key role in enhancing corporate new quality productive forces. This can be explained as follows: industrial upgrading promotes technological innovation and knowledge accumulation, incentivizing enterprises to increase R&D investment, thereby enhancing technological levels and production efficiency. This innovation-driven growth model not only strengthens corporate core competitiveness but also significantly enhances new quality productive forces, laying a solid foundation for the long-term sustainable development of enterprises.

In summary, industrial park-driven industrial upgrading significantly enhances enterprises' new quality productive forces through a dual mechanism of reducing the probability of informed transactions and increasing R&D investment. This finding not only validates the positive role of industrial upgrading in promoting high-quality enterprise development but also provides important practical insights for policymakers and enterprise managers.

### 4.5 Moderating effect

The results of the moderating effect test are shown in Table 7. In Column (1), the coefficient of *IUI*_*i, t*_ is 7.909, which is significant at the 0.01 level, and the coefficient of *IUI*_*i, t*_× *NIP*_*i, t*_ is 0.005, which is also significant at the 0.01 level. This indicates that industrial parks exert a positive moderating effect on the influence of industrial upgrading on enterprises' new productive capacity. This finding is consistent with the research of Statsenko et al. (62), which indicates that industrial parks can enhance new-type productive forces by optimizing resource allocation and accelerating technological innovation, thereby enabling enterprises to withstand market fluctuations and external shocks better.

**Table 7:** Moderating effect analysis result.

**Variables**	**(1)**	**(2)**	**(3)**
	*NPRO*_*i, t*_		
*IUI*_*i, t*_	7.909^***^	9.079^***^	12.660^***^
	(18.61)	(19.91)	(20.63)
*NIP*_*i, t*_		−0.0011^***^	−0.001^***^
		(−8.92)	(−11.18)
*IUI*_*i, t*_× *NIP*_*i, t*_			0.005^***^
			(8.88)
*Controls*	Yes	Yes	Yes
Constant	−27.0002^***^	−28.406^***^	−37.641^***^
	(−16.32)	(−17.05)	(−18.89)
*Province FE*	Yes	Yes	Yes
*Industry FE*	Yes	Yes	Yes
*#Obs*.	21,786	21,786	21,786
*R* ^2^	0.207	0.208	0.216

This table reports the results of moderating effect analysis. The first stage of the regression model is: *NPRO*_*i, t*_ **=** α**+**β_1_*IUI*_*i, t*_**+**β_*k*_*Control*_*i, t*_+γ_*i*_+μ_*i*_+ε_*i, t*_, the second stage of the model is: *NPRO*_*i, t*_ **=** α**+**β_1_*IUI*_*i, t*_**+**_β_2_*NIPi, t*_**+**β_*k*_*Control*_*i, t*_+γ_*i*_+μ_*i*_+ε_*i, t*_, and the third stage of the model is: *NPRO*_*i, t*_ **=** α**+**β_1_*IUI*_*i, t*_**+**_β_2_*NIPi, t*_**+***IUI*_*i, t*_× *NIP*_*i, t*_**+**β_*k*_*Control*_*i, t*_+γ_*i*_+μ_*i*_+ε_*i, t*_, where *NIP*_*i, t*_ is the industrial park location data, *NPRO*_*i, t*_ is the new quality productivity level of company i in year t; *Controls*_*i, t*_ indicates control variables in year t, including book-to-market ratio (*BM*_*i, t*_), return on assets (*ROA*_*i, t*_), financial leverage ratio (*LEV*_*i, t*_), firm age (*AGE*_*i, t*_), firm size (*SIZE*_*i, t*_), sales growth rate (*ORG*_*i, t*_), institutional investor ownership ratio (*INI*_*i, t*_), major shareholder holding ratio (*FIR*_*i, t*_). γ_*t*_ and μ_*i*_ denote the province and industry fixed effects, respectively. ε_*t*_ denotes the residual. The t-values of coefficients are given in parentheses.

^***^ represent significance levels of 0.01, respectively.

Possible explanations for this include the synergistic catalytic effect of the innovation ecosystem. Industrial parks enhance the efficiency of industrial upgrading by establishing a complete innovation ecosystem. The integrated network of “basic research-applied R&D-technology transfer” formed within the park bridges the final gap between technological breakthroughs and industrial applications. This ecosystem enables enterprises to quickly identify application scenarios and supporting resources for technological upgrades, significantly shortening the cycle for realizing innovation value.

Secondly, the intensive effect of factor allocation. The unique characteristics of industrial park aggregation have greatly improved the matching efficiency of innovative factors such as talent, capital, and technology. During industrial upgrading, enterprises can obtain the specialized factor support they need locally, reducing the transaction costs and uncertainties of innovation activities. This intensive allocation model amplifies the marginal output of enterprise R&D investments.

Thirdly, the policy leverage effect of institutional innovation. As a policy testing ground, industrial parks reduce enterprises' institutional transaction costs through institutional innovation. Special regulatory sandboxes, simplified approval processes, and targeted policy support provide enterprises with a more lenient space for innovation and a more efficient policy response mechanism for industrial upgrading, effectively activating their innovation momentum. Fourthly, the multiplier effect of industrial networks. The close interaction among enterprises within the park has created a rapid channel for knowledge spillovers. Geographic proximity has facilitated the dissemination of tacit knowledge, industrial chain linkages have accelerated innovation diffusion, and competitive pressures have driven continuous improvement. This network effect enables the industrial upgrading of individual enterprises to generate a multiplier effect far exceeding their scope, forming a systemic enhancement where “1 + 1 > 2.”

This regulatory effect fundamentally alters the production function of innovation activities through spatial restructuring. As a special institutional space and organizational form, industrial parks reconfigure the combination of innovation elements, optimize the institutional environment for innovation activities, and improve the pathways for realizing innovation value. This enables enterprises to invest in industrial upgrading to generate greater improvements in new productive capacity.

### 4.6 Heterogeneity analysis

#### 4.6.1 Property rights nature

Differences in the ownership structure of enterprises are key factors influencing corporate behavior and performance. Different property rights structures result in significant differences in enterprises' resource acquisition capabilities, innovation incentives, and strategic objectives (63), which may lead to varying effects on the enhancement of new productive forces during industry upgrading driven by industrial parks among enterprises with different ownership structures. Based on this, we divided the sample into two groups: state-owned enterprises and non-state-owned enterprises. We conducted separate regression analyses to examine the moderating effect of property rights structure.

Columns (1) and (2) of Table 8 report the effects of industrial upgrading on new-type productive capacity for state-owned and non-state-owned enterprises, respectively. The results show that in the non-state-owned enterprise sample, industrial upgrading has a significantly positive effect on enterprise new-type productive capacity, and this effect is significant at the 1% level. In the state-owned enterprise sample, while the significance is also significant at the 1% level, the promotional effect is weaker and relatively lower compared to non-state-owned enterprises. This suggests that, compared to state-owned enterprises, non-state-owned enterprises achieve more pronounced improvements in new-type productive capacity during industrial upgrading driven by industrial parks.

**Table 8:** Firm heterogeneity analysis.

**Variables**	**(1)**	**(2)**	**(3)**	**(4)**	**(5)**	**(6)**	**(7)**	**(8)**	**(9)**
	**Ownership**		**Environmental violation incident**		**Low-carbon pilot cities**		**Region**		
	**State-owned**	**Non state-owned**	**Yes**	**No**	**Yes**	**No**	**EC**	**CC**	**WC**
*IUI*_*i, t*_	6.708^***^	7.849^***^	0.199	7.959^***^	10.450^***^	6.322^***^	10.727^***^	7.518^***^	5.381^***^
	(11.05)	(13.09)	(0.13)	(18.72)	(14.86)	(11.23)	(16.30)	(9.52)	(6.75)
*Controls*	Yes	Yes	Yes	Yes	Yes	Yes	Yes	Yes	Yes
Constant	−23.042^***^	−25.313^***^	−2.379	−27.200^***^	−34.738^***^	−19.570^***^	−36.655^***^	−22.256^***^	0.000
	(−10.14)	(−10.22)	(−0.25)	(−16.40)	(−13.83)	(−9.32)	(−15.62)	(−6.38)	0.86
*Province FE*	Yes	Yes	Yes	Yes	Yes	Yes	Yes	Yes	Yes
*Industry FE*	Yes	Yes	Yes	Yes	Yes	Yes	Yes	Yes	Yes
*#Obs*.	10,575	11,211	177	21,609	13,459	8,327	14,780	3,026	2,891
*R^2^*	0.200	0.236	0.224	0.208	0.201	0.237	0.225	0.233	0.239

This table reports the results of heterogeneity analysis based on the ownership, Environmental violation incident, Low-carbon pilot cities, geographical location, EC, CC, WC represent the regions of East, Central, West of China respectively. The baseline regression is: *NPRO*_*i, t*_ = α+β_1_*IUI*_*i, t*_+β_*k*_*Control*_*i, t*_+γ_*i*_+μ_*i*_+ε_*i, t*_, where *NPRO*_*i, t*_ is the new quality productivity level of company i in year t; *Controls*_*i, t*_ indicates control variables in year t, including book-to-market ratio (*BM*_*i, t*_), return on assets (*ROA*_*i, t*_), financial leverage ratio (*LEV*_*i, t*_), firm age (*AGE*_*i, t*_), firm size (*SIZE*_*i, t*_), sales growth rate (*ORG*_*i, t*_), institutional investor ownership ratio (*INI*_*i, t*_), major shareholder holding ratio (*FIR*_*i, t*_). γ_*t*_ and μ_*i*_ denote the province and industry fixed effects, respectively. ε_*t*_ denotes the residual. The t-values of coefficients are given in parentheses.

^***^ represent significance levels of 0.01, respectively.

A possible reason for this difference is that non-state-owned enterprises typically face stronger market competition pressures and respond more swiftly to the technological changes and market opportunities brought about by industrial upgrading. They are more inclined to proactively adopt new technologies and optimize production processes, thereby enhancing total factor productivity and green innovation levels. In contrast, state-owned enterprises may be constrained by institutional barriers, with longer innovation decision-making processes and relatively slower responses to industrial upgrading. Additionally, non-state-owned enterprises can more flexibly adjust resource allocation within industrial parks, such as quickly introducing advanced equipment, optimizing human capital structures, or collaborating with universities and research institutions within the park, thereby more efficiently converting industrial upgrading into productivity improvements. State-owned enterprises, however, may suffer from lower resource allocation efficiency due to administrative intervention or policy-related burdens, thereby weakening the effects of industrial upgrading. Furthermore, state-owned enterprises typically enjoy more government subsidies and policy support, which may reduce their reliance on the innovation environment within industrial parks, thereby diminishing the marginal effects of industrial upgrading. Non-state-owned enterprises, on the other hand, are more dependent on the innovation ecosystem within industrial parks, so the promotional effects of industrial upgrading on their new quality productive forces are more pronounced.

#### 4.6.2 Environmental violations

In recent years, with the deepening of green and low-carbon development concepts (64), a company's environmental compliance performance has increasingly become a key factor influencing its sustainable development capabilities. In this context, as an important vehicle for industrial upgrading, the relationship between industrial park environmental governance levels and companies' new quality productive forces warrants further exploration. This study uses regression analysis based on whether companies have incurred environmental violations to examine the moderating effect of environmental compliance performance on the outcomes of industrial upgrading.

Columns (3) and (4) of Table 8 report the impact of industrial upgrading on enterprises' new quality productive forces for the group without environmental violations and the group with environmental violations, respectively. The results show that in the group without environmental violations, industrial upgrading has a significant positive effect on new quality productive forces; however, in the group with environmental violations, the coefficient for industrial upgrading is positive but not significant. This suggests that, compared to enterprises with environmental violations, industrial upgrading in environmentally compliant enterprises has a more significant effect on enhancing new quality productive forces. The possible reason is that enterprises without environmental violations typically have more substantial environmental protection technology reserves and green management systems, enabling them to convert industrial upgrading into actual productivity more efficiently. Some leading enterprises have achieved resource utilization of waste materials through circular economy pilot programs in industrial parks, significantly reducing production costs. In contrast, companies with environmental violations may struggle to benefit from industrial upgrading due to insufficient environmental investments. On the other hand, industrial parks often provide preferential support to companies with outstanding environmental performance. Environmentally compliant companies can thus more smoothly access innovative resources, amplifying the effects of industrial upgrading. In contrast, companies with environmental violations may face constraints such as environmental production restrictions and limited financing, weakening their upgrading momentum. Finally, in high-standard carriers such as ecological parks, environmentally compliant enterprises are more likely to be embedded in green supply chain networks. For example, a new energy vehicle manufacturer leveraged the industrial cluster effect of an industrial park to form a closed-loop collaboration with upstream battery recycling enterprises, thereby multiplying the efficiency gains from industrial upgrading. In contrast, environmentally non-compliant enterprises may be excluded from high-end supply chains and unable to benefit from collaborative innovation.

#### 4.6.3 Low-carbon pilot cities

Low-carbon pilot cities and non-low-carbon pilot cities exhibit systemic differences in policy frameworks, industrial structures, and innovation environments (44, 45). These differences may result in significant regional heterogeneity in the promotional effects of industrial parks on new productive forces during industrial upgrading processes. Grouping and regression analysis based on whether a city is a low-carbon pilot city can help examine the moderating effects of policy environments on industrial upgrading outcomes.

Columns (5) and (6) of Table 8 present the impact of industrial upgrading on enterprises' new productive forces in low-carbon pilot cities and non-low-carbon pilot cities, respectively. In the low-carbon pilot city group, the coefficient estimate for industrial upgrading is 10.450 and is significant at the 1% level; in the non-low-carbon pilot city group, the coefficient is 6.322, which is also significant but has a relatively weaker impact. This indicates that, compared to non-low-carbon pilot cities, industrial parks in low-carbon pilot cities have a more significant role in enhancing new quality productive forces through industrial upgrading. The reasons are as follows: Low-carbon pilot cities typically have more comprehensive policy support systems, enabling their industrial parks to integrate low-carbon transition policies with industrial upgrading strategies. Some pilot parks use policy tools such as carbon emissions trading and green technology subsidies to significantly reduce the costs of clean technology innovation for enterprises, thereby amplifying the productivity-enhancing effects of industrial upgrading. Industrial parks in low-carbon pilot cities often form more vibrant green innovation ecosystems. Taking a low-carbon industrial park in Shenzhen as an example, the park has gathered specialized institutions such as carbon neutrality technology laboratories and green financial service centers, providing technical support and financial guarantees for corporate low-carbon transformation, enabling the innovative outcomes of industrial upgrading to be more quickly converted into actual productivity. Additionally, industrial parks in low-carbon pilot cities place greater emphasis on building circular economy supply chains. Research shows that the Suzhou Industrial Park has established an inter-enterprise network for energy cascading utilization and waste resource recovery, resulting in an average energy efficiency improvement of 25% for enterprises participating in industrial upgrading, significantly higher than the 15% average in non-pilot city industrial parks.

#### 4.6.4 Regional differences

Regional economic development levels are one of the key factors influencing the implementation effectiveness of industrial park policies. Enterprises in different regions exhibit significant differences in terms of industrial foundations, innovation resources, and policy support (65). These differences may lead to regional variations in the promotional effects of industrial upgrading driven by industrial parks on enterprises' new-quality productive capacity. Through regional heterogeneity analysis, the spatial differences in industrial upgrading effects can be thoroughly examined, providing a scientific basis for formulating industrial policies to promote regional coordinated development. To this end, we refer to existing research (66) and divide the sample enterprises into three regional groups—eastern, central, and western—for regression analysis.

Columns (7) to (9) of Table 8 report the impact of industrial upgrading on new quality productive forces across different regions. The results show that the industrial upgrading coefficient for enterprises in the eastern region is 10.727, 7.518 for the central region, and 5.381 for the western region, all of which are statistically significant at the 1% level. This indicates that the promotional effect of industrial park-driven industrial upgrading on new quality productive forces exhibits a distinct “east-central-west” gradient decrease. The formation of this regional disparity primarily stems from the following factors: industrial parks in the eastern region typically possess more complete industrial chain support systems and more advanced industrial structures. Additionally, industrial parks in the eastern region have concentrated a large number of universities, research institutions, and innovation platforms. Governments in the eastern region demonstrate greater foresight and continuity in the implementation of industrial policies. The more developed market system in eastern regions provides strong support for industrial upgrading. Taking the Pearl River Delta as an example, the activity levels of technology transaction markets and talent markets within its industrial parks are significantly higher than in other regions, accelerating the flow and allocation of innovative elements.

## 5 Conclusions

This paper constructs an industrial park development indicator system based on data from Chinese listed companies from 2000 to 2023. It uses econometric analysis methods to examine the mechanism by which industrial upgrading driven by industrial parks affects corporate new quality productive forces. The findings are as follows: (1) Industrial upgrading driven by industrial parks significantly enhances the level of new-quality productive capacity in enterprises; (2) Mechanism analysis indicates that industrial upgrading can enhance the level of new-quality productive capacity in enterprises by reducing the probability of informed transactions and increasing R&D investment; (3) Moderating effects reveal that the agglomeration effect of industrial parks significantly promotes the enhancement of new-quality productive capacity levels in enterprises through industrial upgrading; (4) Heterogeneity analysis reveals that industrial upgrading has a more substantial and more significant promotional effect on the level of new quality productive forces for enterprises without environmental violations; non-state-owned enterprises and those located in low-carbon pilot cities exhibit a more pronounced promotional effect of industrial upgrading on new quality productive forces; regional difference analysis shows that the effects of industrial upgrading exhibit a “east-central-west” gradient decrease.

This study not only expands the research dimensions of the new quality productive forces theory but also provides empirical evidence for the high-quality development of industrial parks. The findings reveal that industrial parks, as essential carriers of innovation ecosystems, effectively promote the transformation of industrial upgrading into new quality productive forces through mechanisms such as spatial agglomeration, institutional innovation, and policy coordination. This discovery provides important references for governments at all levels to formulate differentiated park development policies and for enterprises to choose upgrading and transformation paths, as well as a new analytical framework for investors to assess regional industrial development potential.

Based on the research conclusions, this paper proposes the following policy recommendations: First, in terms of strategic layout, enterprises should prioritize industrial parks with complete supporting facilities and sound innovation ecosystems for relocation, fully leveraging park resources to enhance innovation capabilities. In terms of transformation and upgrading, enterprises should align with the industrial policy orientation of the park and focus on breaking through key core technologies. In terms of collaborative innovation, enterprises should deeply integrate into the park's innovation network and establish stable cooperative relationships with universities and research institutions.

Second, optimize service provision by establishing a multi-tiered service system comprising “basic services + value-added services + specialized services.” For example, parks can offer value-added services such as technology transfer and talent training. Strengthen the innovation ecosystem by prioritizing the innovation echelon comprising “science and technology-based small and medium-sized enterprises, high-tech enterprises, and leading enterprises.” Promote green development by establishing a park-level carbon emissions monitoring platform and carbon asset management system.

Third, differentiated support policies should be implemented by developing park upgrading roadmaps tailored to the distinct developmental stages of eastern, central, and western regions. Improve the evaluation indicator system and incorporate indicators related to new productive forces into park performance evaluations. Simultaneously, regional coordination mechanisms should be established to facilitate the free flow of innovative elements between parks. Cross-regional industrial innovation alliances can be established to achieve shared R&D resources and mutual recognition of scientific and technological achievements.

This study has the following limitations: (1) the concept of new-type productive forces was proposed relatively recently; hence, the sample period selected for this study spans 2012–2022. Future research may consider extending the sample period to capture the long-term effects of new-type productive forces development. (2) While this study examines the pathways through which industrial upgrading influences the development of new-quality productive forces via informed transaction probability and R&D investment, it does not assess the robustness of these findings. Future research may strengthen this section with additional empirical evidence. (3) Variables such as regional policy support at the industry level and a firm's position within its supply chain may significantly moderate the effect of industrial upgrading on a firm's new-quality productive forces. Future studies could incorporate external environmental variables for a multi-level analysis.

## Data Availability

The original contributions presented in the study are included in the article/supplementary material, further inquiries can be directed to the corresponding author.
